# Challenges and innovations in CAR-T cell therapy: a comprehensive analysis

**DOI:** 10.3389/fonc.2024.1399544

**Published:** 2024-06-11

**Authors:** Jingming Luo, Xianwen Zhang

**Affiliations:** College of Bioscience and Biotechnology, Hunan Agricultural University, Changsha, China

**Keywords:** CAR-T therapy, TME, CRS, Boolean logic gate strategy, CAR-NK therapy

## Abstract

Recent years have seen a marked increase in research on chimeric antigen receptor T (CAR-T) cells, with specific relevance to the treatment of hematological malignancies. Here, the structural principles, iterative processes, and target selection of CAR-T cells for therapeutic applications are described in detail, as well as the challenges faced in the treatment of solid tumors and hematological malignancies. These challenges include insufficient infiltration of cells, off-target effects, cytokine release syndrome, and tumor lysis syndrome. In addition, directions in the iterative development of CAR-T cell therapy are discussed, including modifications of CAR-T cell structures, improvements in specificity using multi-targets and novel targets, the use of Boolean logic gates to minimize off-target effects and control toxicity, and the adoption of additional protection mechanisms to improve the durability of CAR-T cell treatment. This review provides ideas and strategies for the development of CAR-T cell therapy through an in-depth exploration of the underlying mechanisms of action of CAR-T cells and their potential for innovative modification.

## 1 Introduction

Malignant tumors represent a serious threat to human health. Malignancies result from the unrestricted proliferation and spread of abnormal cells. These cells are highly invasive and can reach distant parts of the body through the blood or lymphatic system to form metastatic foci which can impair organ function and not only jeopardize the health and quality of life of patients but may also result in death (1). For many years, traditional treatments, such as surgery and radiotherapy, have been used for treating cancers; however, these methods have certain limitations, particularly in terms of side effects which frequently adversely affect the patient’s quality of life. According to the findings of the GLOBOCAN 2018 Cancer Incidence and Mortality Estimates prepared by the International Agency for Research on Cancer (IARC), lung cancer is the most common malignancy, accounting for 11.6% of total cancer cases, and represents the current number-one killer among cancers, followed by breast, prostate, colon, stomach, and liver cancers, among others (2).

In recent years, rapid advances in oncology, immunology, and molecular biology have led to the development of tumor immunotherapy (3, 4). Immunotherapy has shown durable anti-tumor responses in patients with metastatic cancer. Adoptive cell therapy (ACT) has been shown to induce complete recovery in patients with melanoma. This is based on the principle that endogenous T cells can be genetically modified *in vitro* to specifically target and destroy tumor cells, followed by re-infusion into the patient’s body, resulting in the elimination of the tumor. This approach has now been extended to various cancer types. The immunotargeting of mutant “neoantigens” expressed on tumor cells plays an important role in the success of ACT and other immunotherapies. It also presents new challenges and opportunities for ACT and suggests that sequencing of tumor genomes will identify potential antigens on all tumors (5).

ACT therapy is derived from engineered T-cell therapy. In the 1980s, early ACT therapies were used successfully for the treatment of metastatic melanoma by culturing patient tumor-infiltrating lymphocytes (TIL). However, the use of tumor-infiltrating lymphocyte (TIL) therapy is relatively uncommon due to technical and application limitations. Only a small proportion of TILs are T cells that specifically recognize tumor antigens, and thus large expansion cultures are required. In addition, TIL therapy is used mainly for the treatment of melanoma, and there are no methods available for the successful culture of TILs for other tumors. Thus, there are obvious limitations, and the pursuit of suitable therapies has encountered design and technological bottlenecks on the way to the discovery of alternatives, which has led to the development of ACT therapy.

**Figure 1:**
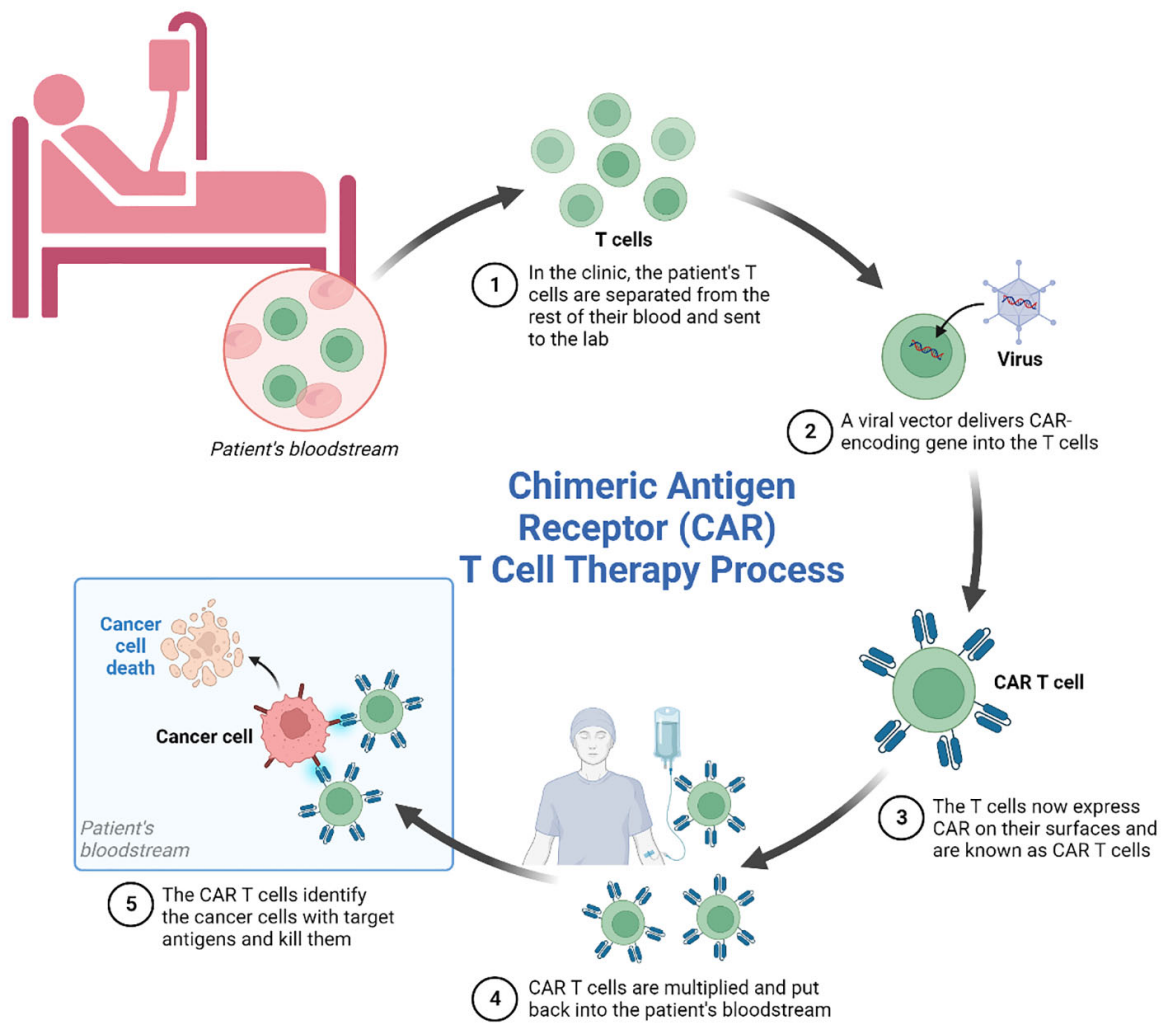
Schematic representation of the main process of CAR-T cell immunotherapy.

Against the technological backdrop of rapid advances in synthetic biology and genetics and cellular engineering, chimeric antigen receptor T cell (CAR-T) therapy, conceived by the genetic engineering of modifications to autologous or allogeneic T cells expressing chimeric antigen receptors (CARs), has emerged as a promising immunotherapeutic approach for various types of cancer. The treatment involves the modification of a patient’s own T cells to express a CAR-That recognizes and binds to specific tumor antigens, leading to the activation and subsequent destruction of the cancer cell. The therapy has evolved rapidly, offering new hope to patients. Nevertheless, CAR-T cell therapy still has some limitations and even side effects, as it is unable to effectively infiltrate solid tumors due to the immunosuppressive effects of the tumor microenvironment. Side effects, such as off-target effects and neurotoxicity, indicate a lack of precision in its design, and this together with cytokine release syndrome, are critical issues associated with the efficacy of the therapy (**Figure 1**).

## 2 CAR-T cell structure

### 2.1 The CAR-T cell principle

Synthetic biology is an emerging interdisciplinary field whose main thrust is the design and construction of new biological parts, devices, and systems, as well as the redesign of pre-existing biological systems. The ultimate goal of synthetic biology is to be able to understand and create biological phenomena at the molecular level.

Advances in genetic engineering and synthetic biology have resulted in a focus on ACT research-engineered T cells, including TCR-T therapy and CAR-T cell therapy. CAR-T cell therapy involves the recognition and binding of CARs to specific antigens on the surfaces of tumor cells by the introduction of CAR genes into the T cells, resulting in a more effective antitumor action (6).

**Figure 2:**
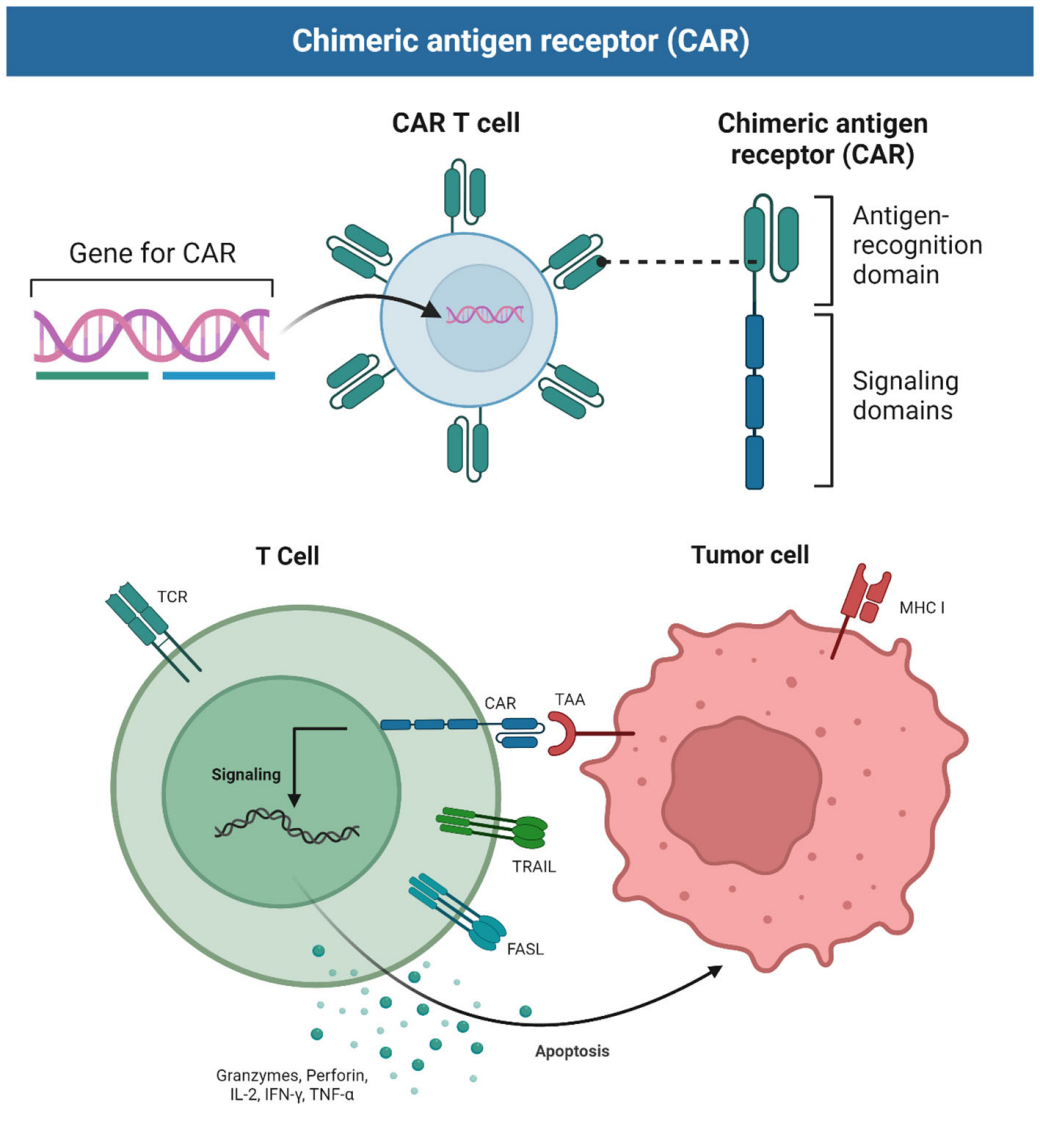
Schematic showing the principles of CAR-T cells.

Structurally, the CAR consists of several parts, namely, a single-chain variable fragment (scFv) antibody, a hinge region, transmembrane structural domains, and an intracellular signaling peptide domain. The scFv component consists of the single-chain variable region of a monoclonal antibody, including both the light chain variable (VL) and heavy chain variable (VH) regions, linked together by a linker region. The function of the scFv is the recognition of tumor-associated antigens (TAAs). The hinge region is located between the scFv component and the structural transmembrane domain, and consists of protein sequences derived from molecules such as CD8 α, TCR β, and IgG. These regions can be deformed and telescoped to provide sufficient folding space for the extracellular antigen-binding region and connect the intracellular region of the molecule to the extracellular region, as well as facilitate approach to the antigen. The transmembrane domain is a structural domain derived from proteins such as CD4, CD7, CD8, CD28, CD137, and CD3ζ, that connects the extracellular and intracellular regions of the CAR and ensures localization and stabilization of the molecule in the T cell membrane. The intracellular signal peptide region, which is usually composed of co-stimulatory molecules (CMs) and immunoreceptor tyrosine-activated modulators (ITAMs), usually TCR/CD3ζ and FcϵRIγ, is responsible for the transmission of the activation signal produced by the interaction of the extracellular T-cell receptor (TCR) region with TAAs to downstream targets within the cell. Thus, CAR-T cells can be activated by binding to target antigens expressed on the tumor cell surface (**Figure 2**). The key difference between CAR-T cells and TCR T cells is that the former do not rely on immunogenic processing and peptide presentation by major histocompatibility complex (MHC) receptors, thus increasing the applicability of CAR-T to different patients (7, 8).

The principle of action of CAR-T cells is that their immune synapses bind to the antigenic targets on the surface of tumor cells, which in turn secretes substances such as perforin and granzyme, causing holes to be created in the cell membranes of the target tumor cells, and then the granzyme enters the target tumor cells through the holes, resulting in a chain reaction of cysteine-aspartate proteases that cause the tumor cells to lysate, and the CAR-T can autocrine release cytokines to promote its own activity and regulate the tumor microenvironment. Alternatively, it can induce apoptosis of target cells via the Fas-FasL pathway through targeted binding of TNF ligand (tumor necrosis factor) on CAR-T cells.

### 2.2 Commonly used targets

The common features of CAR-T cell targets and their respective application time points are comprehensively detailed in **Table 1**.

**Table 1:** Overview of Common Targets of CAR-T Cell Therapy, with Cancer Types and Regulatory Approval Status.

Targets	Suitable tumor types	Approval status
CD19	B-cell lymphoma	FDA approval in 2017 for relapsed or refractory pediatric and adult B-cell precursor acute lymphoblastic leukemia
BCMA	Multiple myeloma	FDA approval in 2021 for treatment of patients with relapsed or refractory multiple myeloma after four or more prior lines of therapy
CD20	Chronic lymphocytic leukemia and other B-cell malignancies	Currently in clinical trials to evaluate effectiveness in treating CLL and other B-cell disorders
CD22	Acute lymphoblastic leukemia	Currently in clinical trials; not yet FDA-approved
HER2	Certain solid tumors	Currently in early clinical trials; not yet approved; focusing on treatment of HER2-positive solid tumors
CD30	Hodgkin lymphoma and others	Currently in clinical trials, focusing on relapsed or refractory Hodgkin and non-Hodgkin lymphomas
CD33	Acute myeloid leukemia	Currently in clinical trials, aimed at treatment of refractory or relapsed AML
CD123	Acute myeloid leukemia and others	Currently in clinical trials, targeting AML and other myeloid malignancies such as myelosarcoma

## 3 The development process of CAR-T cell products and its shortcomings

The results of the first generation of CAR-T cells with scFvs binding to the structural transmembrane domain of CD3ζ suggested the emergence of tumor-specific cytotoxicity (9). However, the *in vivo* anti-tumor efficacies of this generation of CAR-T cells were relatively limited due to because of inadequate persistence of the cells, resulting in insufficient activation and poor cytokine production. The second- and third-generation CAR-T cells incorporate 4–1BB or CD28 co-stimulatory molecules together with CD3ζ, significantly enhancing the anti-tumor effects of the cells, as well as the persistence and proliferation of the CAR-Ts *in vivo* (10). The fourth generation of CAR-T cells introduced additional co-stimulatory ligands, such as CD28, CD137, or CD134 on the basis of the success of the previous generations (11). Compared with its predecessors, the killing ability of the fourth-generation cells was further enhanced by the introduction of cell suicide genes, such as HSV1-tk or iCaspase9, thus improving the precise regulation of CAR-T cell activity while reducing toxic side effects through drug modulation. The fifth generation of CAR-T cells focused on the development of universal CARs as allogeneic CAR-T cell therapies, which can eliminate GvHD (Graft-versus-Host Disease) and MHC-mediated host-versus-graft reaction HVGR (Host-versus-graft reaction) by the knockdown of endogenous TCR, MHC, and genes encoding other signaling proteins on the T-cell surface through gene editing technology (12). In addition, to improve the survival and cytotoxicity of CAR-T cells *in vivo*, T-cell suppressor signaling molecules, such as PD-1 and CTLA4, were knocked down and the T-cell signaling region of the antigen-targeting domains was disassembled, allowing the recognition and binding of additional antigens. This strategy provides both a broader perspective for the application of CAR-T cells and new ideas for the treatment of patients showing poor activity in their isolated T cells, which is an obstacle to genetic modification.

**Figure 3:**
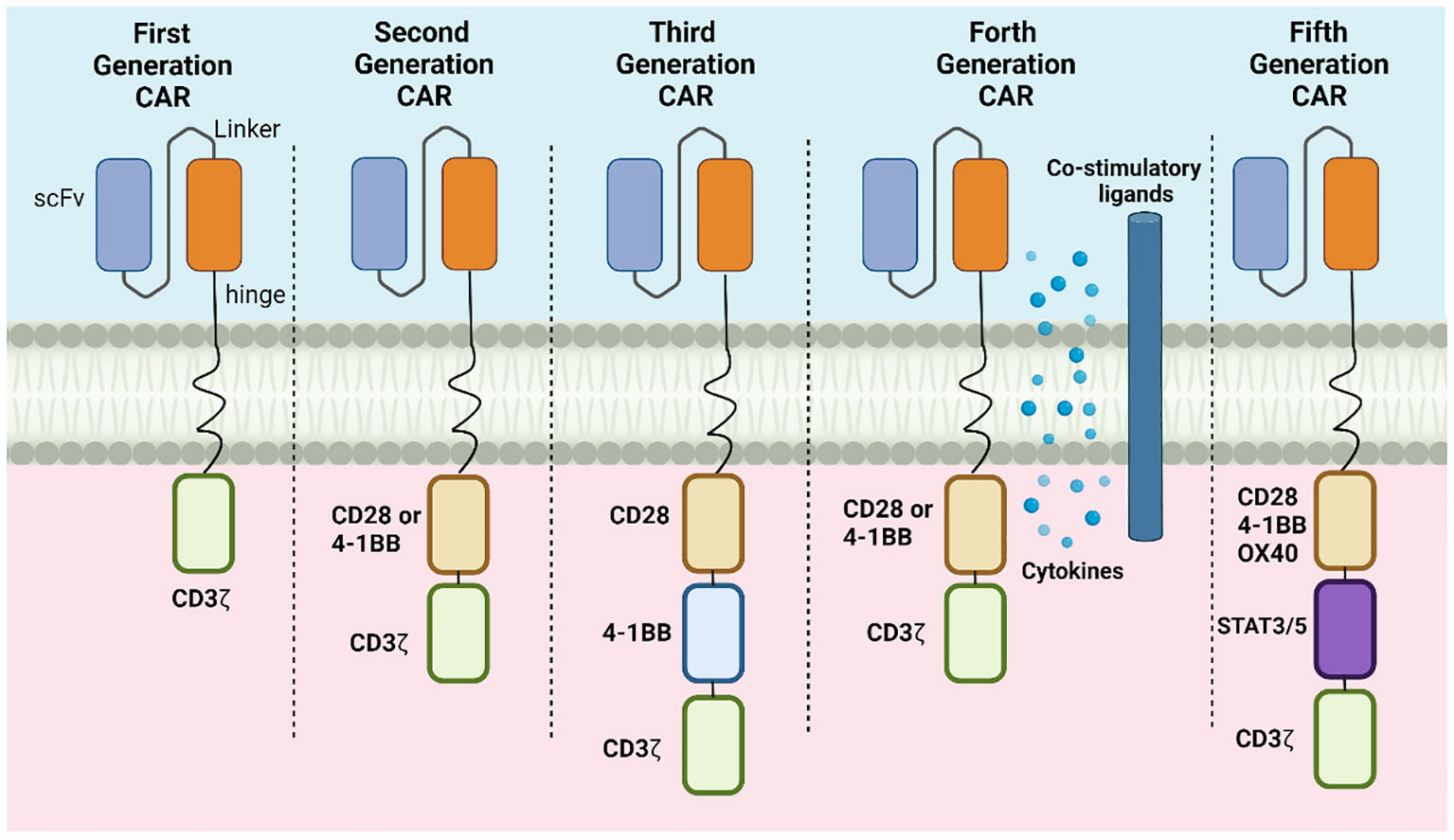
The development of the CAR-T cell design, showing changes in structures.

CRISPR/Cas9 gene editing technology was used in the development of fifth-generation CAR-T cells to knock down endogenous TCR and MHC I molecules. However, this has some drawbacks, specifically, in that CRISPR/Cas9 gene editing may result in severe off-target effects. Generally speaking, the development and application of generalized CAR-T cells also have great potential for cost reduction in immunotherapy. An illustration of the structural development is shown in **Figure 3**.

## 4 Challenges associated with the use of CAR-T cells in solid tumors

### 4.1 Overcoming the problem of antigenic heterogeneity to improve targeting precision

Tumor antigen heterogeneity is a common problem in CAR-T cell therapy. Analyzing the expression levels of tumor antigens in tumor tissues showed that there are significant differences between patients with different tumors, which implies that the heterogeneity of a variety of tumor antigens must be taken into account in patient selection and assessment of the therapeutic efficacy of CAR-T cell therapy (13–15). Currently, the most frequently used strategy uses a combination of bispecific antibodies (BsAbs). The use of BsAbs allows the targeting of CAR-T cells to tumors with high specificity, thereby improving tumor targeting and killing. By combining the binding specificities of two antibodies, BsAbs can recognize both CAR-T cells and tumor antigens. The potential of using BsAbs together with CAR-T cells is supported by data-driven evidence. A recent study demonstrated that co-administration of BsAbs targeting CAR-T cells and tumor antigens enhanced both CAR-T cell activation and tumor regression in a mouse model of lymphoma. It was also found that BsAbs promote interactions between the CAR-T cells and tumor cells, thus facilitating the formation of immune synapses, and enhancing the cytotoxic effects of CAR-T cells against tumor cells. The use of BsAbs also broadens the range of target choices for CAR-T cell therapy and increases the likelihood of tumor elimination (16). However, numerous potential antigenic targets on solid tumors are also expressed in low amounts in normal cells, and the severe toxicity associated with the off-target response represents a significant safety challenge (17).

On the way to overcome the challenge of tumor heterogeneity, some researchers explored the effect of tumor heterogeneity on the function of MSLN-CAR-T (Mesothelin, MSLN) cells and searched for strategies to overcome this effect. The investigation demonstrated that CD28 co-stimulated MSLN-CAR-T cells (M28z) had better killing capacity relative to 4–1BB co-stimulated MSLN-CAR-T cells (MBBz). Emphasis was placed on the fact that CD28 co-stimulated MSLN CAR-T cells had a greater killing ability against tumor spheroids expressing heterogeneous MSLN compared to MBBz CAR-T cells (18). This study provides some ideas and strategies to address tumor heterogeneity.

### 4.2 CAR-T cell therapy and tumor infiltration

The tumor microenvironment (TME) is a complex ecosystem that includes tumor, immune, and, mesenchymal stromal cells, as well as blood vessels and molecular components. It presents a barrier surrounding the tumor cell and has an important impact on the therapeutic efficacy of CAR-T cells. Studies have shown that immunosuppressive factors in the TME, such as immune checkpoint proteins, suppressor cytokines, and regulatory T cells, limit the activation and proliferation of CAR-T cells. In addition, solid tumors tend to have high cell densities and low vascular densities, restricting the infiltration and spread of CAR-T cells and thus potentially reducing their effectiveness against the tumor (19–21).

Researchers have long recognized this problem and have designed strategies that can overcome the inhibitory effect of the TME on CAR-T cells, specifically, by using a combination of immune checkpoint inhibitors and CAR-T cell therapy and thus enhancing the therapeutic efficacy of the CAR-T cells. A study showed that the combined application of programmed cell death protein-1 (PD-1) inhibitors and CAR-T cell therapy achieved good therapeutic effects in a solid tumor mouse model, significantly improving both the survival and infiltration capacity of the CAR-T cells (22). In addition, the use of gene editing techniques to modify CAR-T cells to overcome the restrictions of the TME is also a potential solution. It has been found that the introduction of genes that enhance sensitivity to antitumor drugs can enhance the cytotoxic abilities of CAR-T cells even in the presence of high concentrations of chemotherapeutic drugs (23). Currently, researchers expect to block the binding between tumor cells and immune checkpoints using PD-1/PD-L1 inhibitors to prevent immune escape; however, the results of clinical trials have not been promising as patients showed adverse effects (24). In layman’s terms, the immune checkpoints themselves act as “brakes” that require activation to mitigate the development of severe side effects. In addition, some researchers have used the Siglec15 protein to determine the therapeutic effects against lung adenocarcinoma in nude mice. It was found that an anti-Siglec15 antibody enhanced the treatment of lung adenocarcinoma with increased destruction of tumor cells, possibly due to macrophage polarization affecting the TME. Both *in vitro* and *in vivo*, cMet/Siglec15 CAR-T cells showed obvious cytotoxicity against lung adenocarcinoma cells, and at the same time, they could significantly inhibit the growth of lung adenocarcinoma cells *in situ* and increase the infiltration of T cells in the TME (25). The study also showed that cells expressing CXCL9 enhanced these effects. In another study, the use of CXCL9 was shown to increase T-cell infiltration into tumors and also inhibit tumor angiogenesis. The researchers designed mesothelin-targeted CAR-T cells (CARmeso-CXCL9) that expressed CXCL9, and demonstrated that these CARmeso-CXCL9 cells increased both the migration capacity and cytotoxicity of CAR-T cells in both cellular and animal models (26).

A recent study armed a lysosomal adenovirus (OADs) with the chemokine CXCL11 to increase CAR-T cell penetration and reprogram the immunosuppressed TME to enhance the therapeutic efficacy. In an immunodeficient and immunoreactive *in situ* GBM (glioblastoma) mouse model, B7H3-targeted CAR-T cells showed a durable anti-tumor response after intratumoral administration in combination with CXCL11-OADs. In addition, CXCL11-OADs remodeled the immunosuppressive TME in the GL261GBM model, resulting in increased infiltration of CD8+ T lymphocytes, natural killer (NK) cells, and M1-polarized macrophages, and a decreased proportion of myeloid-derived suppressor cells (MDSCs), regulatory T cells (Tregs), and M2-polarized macrophages (27).

In a broader sense, durability and safety have always been extremely important concerns for this therapy. Recent research discovered that TGF-β plays a crucial role in immunosuppression by stimulating cytokine synthesis through the NF-κB pathway. Conversely, SMAD7, a negative regulator of TGF-β signaling, helps mitigate CAR-T cell depletion and reduce cytokine production. These modified CAR-T cells demonstrated superior proliferation and reduced depletion markers, indicating potent tumor suppression and decreased cytokine release, lowering the risk of CRS. This strategy suggests a promising approach to improving CAR-T cell effectiveness and safety (28).

### 4.3 Obstacles to trafficking

Trafficking of CAR-T cells in the specific conditions of the TME represents a significant therapeutic challenge. To address this, researchers have recently investigated the role of LIGHT/tumor necrosis factor superfamily member 14 (TNFSF14) in overcoming the barrier to CAR-T cell migration within the TME. Various *in vitro* and *in vivo* experimental methods have been employed to assess the impact of LIGHT/TNFSF14 on CAR-T cells. For example, a simulated TME was created *in vitro* to observe the effects of LIGHT/TNFSF14 on CAR-T cell migration and cytotoxicity. Additionally, an immunodeficient NSG mouse model was utilized to confirm the influence of LIGHT/TNFSF14 on CAR-T cell activity *in vivo*. The results demonstrated that LIGHT/TNFSF14 could significantly enhance CAR-T cell migration within the simulated TME, leading to improvements in both *in vitro* migration and *in vivo* infiltration (29). To further elucidate the migratory capacity of CAR-T cells, researchers developed a radionuclide labeling technique for imaging, using both direct cell labeling and reporter gene strategies for real-time monitoring and tracking. A novel reporter gene, truncated prostate-specific membrane antigen (CAR-PSMA), was constructed and incorporated into a lentiviral vector together with the CAR expression gene to ensure a consistent reporter gene to CAR ratio without compromising the CAR-T cell cytotoxic function. Experimental findings verified that the truncated PSMA was expressed normally in cell membranes, enabling the effective detection of CAR-T cells through binding to its radiolabeled ligand (30).

### 4.4 Inconsistent homing ability

The homing ability is one of the core functions of CAR-T cells, as this determines the ability of the cells to circulate in the body and find and bind to their targets in tumor tissues. Several studies have shown that CAR-T cells have inconsistent homing abilities when used for treating solid tumors. This may be related to several factors. The first of these is the heterogeneity of tumor cells. It has been found that the overall population of tumor cells contains different subpopulations, and CAR-T cells may be more likely to recognize and kill certain subpopulations, leading to the continued growth of the other subpopulations (31). Secondly, the development of fibrosis and blood vessel formation in tumor tissues can also limit the homing ability of CAR-T cells (32), indicating the complexity of the issue of CAR-T cell homing (33). To solve the homing problem, some researchers have constructed a novel dual-targeted tandem CAR-T cell, designed with the basic framework of third-generation CAR-T cells and packaged with uPAR and HER2 antigens by lentiviral transduction. The use of these constructed uPAR/HER2-CAR-T cells resulted in smaller mean volumes of implanted tumors, together with reduced mean mass, a higher number of CAR copies in tumor tissues, a higher number of CD3+ cells, and a higher number of blood tumor cells compared with the conventional CAR-T cell treatment, as well as the uPAR-CAR-T cell and the HER2-CAR-T cell groups. Increased levels of tumor-killing cytokines were also observed in the blood, demonstrating that uPAR/HER2-CAR-T cells could effectively overcome the problem of poor homing (34).

### 4.5 CAR-T therapy triggers CRS and ICANS and strategies to deal with them

CAR-T cell therapy can nevertheless trigger more serious toxicities when treating solid tumors, mainly including cytokine release syndrome (CRS) and immune effector cell-associated neurotoxicity syndrome (ICANS). CRS is an inflammatory response caused by the massive production and release of cytokines by immune cells. Cytokines are a class of protein molecules with multiple biological functions and include tumor necrosis factors (TNFs) and interleukins (ILs). While cytokines have important roles in regulation and signaling during the immune response, their excessive production and release can lead to severe inflammatory responses and tissue damage. Clinical manifestations include hyperthermia, hypotension, tissue edema, and in severe cases, respiratory distress syndrome and multi-organ failure. A study by Lakomy et al. concluded that the onset and severity of CRS were associated both with the tumor load and CAR-T cell dose and that aggressive lymphocyte removal regimens increased the likelihood of CRS (35). ICANS, on the other hand, usually manifests as a variety of symptoms indicative of neurological impairment, such as headache, fatigue, blurred consciousness, and memory loss. The mechanism responsible for its occurrence is not fully understood, but usually involves systemic inflammation resulting from CRS, impaired functioning of the blood-brain barrier, and direct neurotoxic effects. Studies have shown that ICANS can occur concurrently or independently of CRS, and that high-grade ICANS is usually strongly correlated with high-grade CRS, which may have serious implications for the patient’s health (35). Treatment of ICANS usually requires immunosuppressive agents to preserve the patient’s neurological function and vital signs. Recent strategies aim to improve the management of these toxicities. Tocilizumab, a monoclonal antibody targeting the IL-6 receptor, has become the standard of care for CRS for both prophylaxis and early intervention; however, due to its poor penetration of the blood-brain barrier, additional treatment of ICANS is required, usually with corticosteroids (36). One study design used an early combination of dexamethasone and tocilizumab in 43 patients; the ratios of the incidence of high-grade CRS were 0% and 10%, respectively with no worsening of ICANS. In addition, when conventional therapies failed, the IL-1 receptor antagonist anakinra and other emerging therapies showed promise in the treatment of refractory CRS and ICANS, suggesting that anakinra can target specific cytokine pathways to mitigate refractory CRS or ICANS following CAR-T cell therapy for solid tumors (37). Overall, both a full understanding and effective management of CRS with ICANS are critical to improving the safety and efficacy of CAR-T therapy, while the difficulties in this area are extremely challenging due to the complexity of its pathophysiology.

### 4.6 Hemophagocytic syndrome

Hemophagocytic syndrome (HS), also known as hemophagocytic lymphohistiocytosis (HLH), is a rare but potentially fatal symptom of immune dysregulation that manifests as abnormal activation of the immune system with an overreaction of inflammatory cells. Although the occurrence of HS/HLH is uncommon, it is highly significant in CAR-T cell therapy, as this condition can cause serious clinical complications. The etiology of hemophagocytic syndromes is essentially categorized as hereditary or secondary, with the hereditary form originating from autosomal recessive or X-linked inheritance and associated with a clear genetic defect and family history. Secondary disorders are caused by malignant tumors and excess secretion of inflammatory cytokines, triggering overactivation of the immune system. In addition, the expansion and sustained activity of CAR-T cells may also directly or indirectly promote the activation of macrophages and lymphocytes, leading to their misdirected attack and phagocytosis of blood and bone marrow cells, thus causing HS/HLH. The main clinical manifestations of HS/HLH include persistent high fever, enlargement of the spleen and liver, hematopenia (including anemia, leukopenia, and thrombocytopenia), hypertriglyceridemia, hyperglyceridemia, and methemoglobinemia (38). Neurological symptoms, such as spasms or confusion, may also be present, and severe cases are associated with a high mortality rate. This type of CAR-T-related hemophagocytic lymphohistiocytosis-like toxicity (carHLH) is often masked because it is usually comorbid with severe CRS. A recent study investigated differences between carHLH and severe CRS. The results showed significantly elevated levels of IF-γ and IL-6 in both carHLH and severe CRS patients, with significantly higher levels of IF-γ and IL-10 observed in carHLH patients. While IL-6 levels exhibited similar trends in all patients, those of IF-γ peaked at a higher level in carHLH until day 12, declining rapidly thereafter in patients with severe CRS. Additionally, IL-10 levels were significantly higher in patients with carHLH compared to other groups. These markers aid in distinguishing between carHLH and severe CRS, offering valuable insights for carHLH prevention and treatment. In addition, studies have developed a model to predict the development of carHLH using serum lactate dehydrogenase levels on day 6 after the onset of CRS and serum fibrinogen levels on day 3 after CRS onset as indicators (39, 40).

## 5 Challenges in the use of CAR-T cell immunotherapy for hematologic oncology

### 5.1 Off-target effects and neurotoxicity

CAR-T cell therapy has a problem of off-target effects. These result from the fact that CAR-T cells attack both tumor and normal healthy cells, resulting in serious side effects in patients. The expansion and persistence of CAR-T cells *in vivo*, as well as a lack of affinity for tumor cells, may be factors that exacerbate off-target reactions (41). Researchers have found that in CAR-T therapy, a portion of CAR-T cells attack healthy B cells, damaging the patient’s immune system and potentially leading to serious complications such as transfusion dependence (42).

The recognition of normal cells, particularly, nerve cells, can lead to neurotoxic reactions in patients (43). Recent clinical trials have shown that some patients treated with CAR-T cell therapy experience serious adverse reactions, including high fever, lung lesions, and central nervous system damage, among others (44). These adverse reactions are associated with the off-target effects of CAR-T cells. These data clearly indicate that the off-target effects of CAR-T cell therapy are a problem that cannot be ignored. It has also been shown that the neurotoxicity caused by off-target effects can be reduced by targeting co-expressed surface antigen proteins and the use of CRISPR/Cas9 gene editing (45). Another researcher designed a novel inhibitory CAR molecule based on KIR/PD-1, which contains the extracellular portion of killer cell immunoglobulin-like receptor (KIR) and the intracellular portion of PD-1. It was found that T cells co-expressing iKP CAR and CD19 CAR could effectively differentiate between HLA-naïve Daudi cell lines and HLA-expressing normal B cells, while cells of the gastric cancer cell line MGC803, which expresses low levels of HLA, were killed in high numbers, while the effect was markedly reduced in gastric cancer cells expressing high levels of HLA. These results indirectly indicate that the presence of iKP CAR can reduce cytotoxicity toward normal cells with high HLA expression, and the study addresses some of the off-target effects in terms of signaling regulation (46). In terms of specific targeting, another study confirmed that IL-21 can enhance the expansion of T cells and the specific cytotoxicity of CAR-T cells against tumor cells (47). The specific targeting of IL-21 has also been demonstrated by other researchers through the design of comparative trials.

### 5.2 Cytokine release syndrome

The CRS responses are also often seen in hematologic oncology treatments. For treatment, measures such as anti-inflammatory drugs, glucocorticoids, and anti-cytokine antibodies are often used to reduce the inflammatory response and alleviate damage caused by excessive cytokine release. It is hypothesized that after activation and expansion of CAR-T cells *in vivo*, a large number of cytokines (such as tumor necrosis factor-α and interferon-γ) are released, stimulating macrophages to produce even greater amounts of cytokines, triggering a systemic inflammatory response, and ultimately leading to the development of CRS (48). For an in-depth study of the pathogenesis of CRS, several researchers have measured and compared the changes in IL-1β and IL-6 levels in the culture supernatants of macrophages by isolating and culturing normal human peripheral blood mononuclear macrophages, and co-incubating the culture supernatants with CAR-T cells and target cells in which GSDME was knocked out. The results indicated that massive GSDME-mediated tumor cell death was the intrinsic cause of CARS during CAR-T cell therapy. Compared with specific T cells, CAR-T cells release more PRF1/GZMB, which activates the excessive caspase-3 in target cells, thus cleaving GSDME to cause target cell death (49).

Glucocorticosteroids are usually used to reduce and eliminate the toxicity of CRS. Ruxolitinib, formerly known as INCB018424 or INC424, an inhibitor of the IL6/JAK/STAT3 signaling pathway, is effective in mitigating CRS by inhibiting the activation of T cells and CAR-T cells and down-regulating the expression of related cytokines, thereby inhibiting the activation of cytokine receptors (50). This inhibition of cytokine receptors alleviates CRS. Interestingly, the CRS response is often regarded as a marker of successful CAR-T cell therapeutic efficacy in specific immunotherapy treatments, and control of CRS requires graded and precise interventions.

### 5.3 Graft-versus-host disease

Graft-versus-host disease (GvHD) is a serious complication that can occur after allogeneic tissue transplantation, in which the transplanted exogenous bone marrow, peripheral blood stem cells, or lymphocytes perceive the recipient’s body as foreign and trigger an immune response. There are two forms of GvHD, namely, acute and chronic. Acute GvHD usually occurs within the first 100 days after transplantation and manifests in a variety of symptoms, including severe skin rashes, liver dysfunction, and gastrointestinal problems such as nausea, diarrhea, and abdominal pain. Chronic GvHD, on the other hand, can develop later and persist or recur for a long time. To explore the efficacy and safety of CAR-T therapy in the treatment of relapsed/refractory hematological malignancies, as well as the effective prevention of GvHD in the absence of conventional conditioning chemotherapy, a trial of CD7 CAR-T therapy followed by haploidentical hematopoietic stem cell transplantation (Haplo-HSCT) was conducted in patients with CD7-positive acute leukemia. The study excluded conditioning regimens or GvHD prophylaxis. Patients who were heavily pre-conditioned and ineligible for standard allogeneic HSCT achieved significant remission post CAR-T therapy. Despite the development of grade 2–3 CRS, GvHD was managed with steroids and immunosuppressive agents. Several patients achieved complete donor chimerism post-transplantation, indicating successful grafting (51). A further study designed a UCART20x22 double-allogeneic CAR-T therapy, the first-in-human study of UCART20x22 targeting CD20 and CD22 in non-Hodgkin’s lymphoma (NHL). The results showed that all the treated patients responded to therapy with complete or partial remission and significant amplification of UCART20x22. The treatment was well-tolerated, with manageable levels of CRS and no ICANS or GvHD (52). These studies provide promising insights into the use of CAR-T therapy for the treatment of refractory hematologic malignancies. By bypassing conventional chemotherapy for GvHD prophylaxis, this approach not only reduces the toxicity of high-dose CAR-T therapies but also preserves the anti-tumor efficacy of CAR-T cells. Future strategies may focus on the optimization of CAR-T constructs and dosing regimens to further reduce the risk of GvHD while improving therapeutic efficacy. **Table 2** shows the impact of some classical CAR-T production methods on GvHD and features of CAR-T cell preparation.

**Table 2:** Effects of different methods of CAR-T cell preparation on GvHD.

Method	Feature	Cost	Date
VST	No need for gene editing, no risk of off-targeting, milder immune response; low efficiency, poorer long-lasting efficacy, large individual differences	low	1990s
ZFN	GvHD can be avoided completely and effectively, high gene editing efficiency; prone to higher cytotoxicity and risk of gene off-targeting	high	1996
shRNA	Reversible knockdown of the TCR gene with controllability and efficiency, with the possibility of incomplete gene silencing	low	2000s
ARCUS	Completely prevents GvHD, highly specific, more stable edited genome, lower off-target activity	high	2010
TALEN	GvHD can be prevented completely and effectively, with high specificity and precision; some risk of gene off-targeting	high	2011
CRISPR	GvHD can be completely prevented with high precision and the ability to personalize and customize modified T cells; off-target effects and safety issues and unfavorable immune responses are present	high	2012

VST, Virus-Specific T cells; ZFN, Zinc Finger Nucleases; shRNA, short hairpin RNA; TALEN, Transcription Activator-Like Effector Nucleases; CRISPR, Clustered Regularly Interspaced Short Palindromic Repeats.

## 6 Future development strategies for CAR-T cell immunotherapy

### 6.1 Structural and functional modifications of CAR-T cells

The single-target CAR-T cell therapeutic regimen has several defects. (1) The problem of uneven antigen expression on the surfaces of tumor cells, i.e., tumor cell heterogeneity, limits the effectiveness of CAR-T cell therapy in targeting heterogeneous tumor cell populations. (2) The target antigen can change or disappear due to mutation, resulting in tumor cell escape. (3) Normal cells expressing the target antigen are also attacked, resulting in severe toxic side effects. Between 30 and 50% of patients who receive a single-target regimen such as CD19-CAR-T cells and are in remission experience disease recurrence (53) This has been found with CD22, BCM, and other therapeutic regimens and has also been observed after two-point CAR-T cell therapy using both CD22 and BCMA targets (54, 55). Therefore, multi-targeted CAR-T programs are preferable.

There are two strategies for the design of multi-target CAR-T cells. The first is to transfer CAR molecules with different scFvs into the same T cell to form a dual/multi-specific parallel arrangement of CAR-T cells, while the other is to express two or more scFvs on a single CAR molecule, which is then transferred into a single T cell to form a dual/multi-specific tandem arrangement of CAR-T cells (56). Multi-targeted CAR-T cells can effectively address the antigen loss that occurs during treatment and can also reduce the incidence of CRS. A phase I trial of CAR-T cells with CD19 and CD22 in young patients with relapsed or refractory B-cell acute lymphoblastic leukemia demonstrated controlled toxicity, with 5 out of 12 patients showing complete remission (57). A similar study recently assessed the efficacy and safety of dual-targeted CAR-T cell therapy in pediatric patients with relapsed/refractory B-cell acute lymphoblastic leukemia (ALL). Results indicate that dual-targeted CAR-T cell therapy is well tolerated and effective in this patient population. Although the sample size was small, no cases of relapse due to antigenic modulation were observed. However, the shorter duration of the dual-targeted CAR-T cell product compared to single-targeted CD19 CAR-T cell products could be attributed to higher CAR expression levels, which result in activation-induced cell death or exhaustion. Efforts are ongoing to enhance the durability of dual-targeted CAR-T cell therapies (58).

The optimal modification of CAR-T cells using a Bispecific T-cell Engager (BiTE) is also possible. The most desirable goal in tumor immunotherapy is obviously the identification of tumor-specific antigens (TSAs) but this is very difficult, and the actual application is still based on tumor-associated antigens (TAAs), which are expressed in both tumor and normal cells, resulting in shortcomings in terms of specificity (59). BiTE has two types of scFv, one of which recognizes antigens on the tumor cell surfaces, generally TAAs and TSAs, and the other that recognizes CD3 on the T-cell surface, thus enhancing the recognition of tumor cells by both CAR and endogenous T cells; this strategy has shown promise for application and R&D (60). Some experiments have constructed bispecific T cell-binding antibodies that can bind CD3 or CD28, and in a humanized mouse model, stimulation of CD3 and CD28 was found to inhibit tumor growth, stimulate the proliferation of memory/effector T cells, and reduce regulatory T cells in non-human primates at a well-tolerated dose.

The double-chain chimeric receptor has a symmetrical structure consisting of two single-chain chimeric receptors connected by a flexible polypeptide chain. This double-chain structure provides both high binding affinity and stability, which can enhance the ability of CAR-T cells to recognize and bind tumor cells. In addition, CAR-T cell therapies using double-chained chimeric receptors have the advantage of long-lasting anti-tumor effects. The novel cell therapy product STAR-T features a TCRαβ-based dual-chain receptor with variable region immunoglobulin heavy and light chains (VH and VL) fused to TCR-Cα and TCR-Cβ, respectively, in contrast to AbTCR based on TCRγδ and the single-chain scFv-based TAC and TRuC receptors. The conformation of STAR resembles the structure of the natural TCR. This receptor possesses both TCR and antigen recognition properties, and its antigen sensitivity is higher than CAR-T cells, which effectively reduces the risk of tumor recurrence due to antigen loss In a variety of solid tumor models. It has been found that the efficacy of STAR-T exceeds that of conventional CAR-T cell therapy (61).

In addition to structural modifications to enhance precision and efficacy, there have also been recent efforts by investigators to overcome GvHD by designing an allogeneic anti-BCMA CAR-T cell therapy known as Allo-715. This aims to eradicate GvHD as well as minimize CAR-T cell rejection. Allo-715 contains an integrated, self-activating third-generation recombinant lentiviral vector and expresses a second-generation anti-BCMA CAR containing an scFv from a human anti-BCMA antibody, together with the intracellular structural domains of 4–1BB and CD3ζ.^43^ Increasing doses of ALLO-715 were administered to 43 patients with r/r multiple myeloma following an anti-ALLO-647-based lymphocyte depletion regimen, resulting in the development of CRS in 24 patients (55.8%), of which only 1 (2.3%) was at least grade 3, while neurotoxicity occurred in 6 patients (14%), with no adverse events of grade 3 and above (62).

In terms of addressing the issue of CAR-T cell depletion in the TMEs of solid tumors, several researchers have recently induced a low differentiation state in CAR-T cells by overexpression of RUNX3, an important regulator of T-cell immunity, which was found to be effective in reducing CAR-T cell depletion when stimulated by antigens. It was also found to reduce cytokine release in *in vitro* experiments, and CAR-T cells that overexpress RUNX3 are safer than conventional CAR-T cells and show no reductions in their anti-tumor effects (63).

### 6.2 Using Boolean logic gates to set up switching systems to improve CAR-T cell selectivity

Immunotherapy is faced with the problem of improving selectivity of CAR-T cells to tumor antigens while reducing off-target interactions to reduce adverse side effects. This requires constant optimization of solutions. Recently, the concept of utilizing Boolean logic gates has emerged as a promising strategy that can regulate the activity of CAR-T cells under specific conditions by programming them to set switches, thereby improving their specificity. Synthetic synNotch receptors can activate CAR expression following recognition of target ligands on cell surfaces and are thus suitable candidates for switching systems (64). Multiple specific signals can be discriminated by using the “AND” gate, thus improving the recognition of cancer cells by CAR-T cells. The connection of multiple antigen recognition domains to the activation domain allows activation of CAR-T cells only when multiple antigens are detected at the same time, thus avoiding non-specific activation by a single antigen (65). The discrimination of any one of multiple antigens can be achieved by using the “OR” gate. The use of multiple antigen-recognition domains connected to the activation domain together with the appropriate switch allows the activation of CAR-T cells when detecting specific antigens, thus enhancing the specificity of the cells (66). Rational design of the CAR structure and the introduction of appropriate logic control elements allow precise manipulation of CAR-T cells (67). Continuous advances in this cutting-edge research have demonstrated the potential of this strategy and provide new ideas and methods for the precise regulation of CAR-T cells.

Recently, researchers have devised a method for engineering CARs in which intracellular proximal T-cell signaling molecules are utilized in place of the traditional CD3 zeta structural domain. Certain proximal signaling CARs, such as ZAP-70 CAR, can activate T cells and eradicate tumors *in vivo* while bypassing upstream signaling proteins, including CD3 zeta. The primary role of ZAP-70 is to phosphorylate LAT and SLP-76, which form a scaffold for signal propagation. This cooperation between LAT and SLP-76 was applied to the design of the logic-gated intracellular network (LINK) CAR, a rapid and reversible Boolean logic and gated CAR-T cell platform that has been shown to out-perform other systems in terms of efficacy and the prevention of on-target and off-tumor toxicity, while expanding the range of molecules targeted by CAR-T cells (68). In recent years, researchers have also designed adapter CAR (AdCAR) systems that combine biotin-labeled adapter molecules with specific linker structures to re-target the AdCAR-T to the corresponding antigen; this is known as the Linker-Label-Epitope and can act as an “AND” gate. AdCAR-T consists of a two-component signaling system based on a split recognition/activation design, in which the labeled AM (adapter molecules) transmits the antigen-recognition signal to activate the T cells via the anti-labeled CAR, allowing for better specificity and effector control, as well as the ability to recognize multiple antigens to prevent tumor escape (69).

### 6.3 Research on armored CAR-T cell programs to improve durability

In the TME, prolonged stimulation of CAR-T cells by antigens can lead to the expression of multiple inhibitory receptors on their surfaces. This will, in turn, lead to a state of T-cell depletion, significantly reducing both the effector and proliferative capacities of the T cells.

The concept of “armored” CAR-T cells thus has considerable potential in immunotherapy. This has been proposed to overcome the limitations of tumor escape and off-target toxicity, and involves equipping cells with additional protective mechanisms together with second/third generation CAR-T cells, including the co-expression of key cytokines, chemokines, or co-stimulatory ligands, to enhance their immunomodulatory effects and anti-tumor efficacy, as well as their durability.

Improved tumor clearance has been observed in preclinical studies. For example, mice treated with “armored” CAR-T cells showed significantly improved tumor regression compared to mice treated with conventional CAR-T cells. These findings were supported by analysis of tumor-infiltrating lymphocytes and cytokine profiling, suggesting the induction of stronger immune response as well as longer persistence of “armored” CAR-T cells (70, 71).

Clinical trials involving CAR-T cell therapies have observed instances of tumor recurrence due to immune escape resulting from, for example, downregulation or loss of the target antigen (72). By equipping CAR-T cells with additional protective mechanisms, the use of “armored” CAR-T cell therapy can overcome these challenges and improve the long-term efficacy of the treatment. The TME is characterized by immunosuppressive factors and limited T-cell trafficking and poses a significant barrier to CAR-T cell therapy. The expression of chemokines or cytokines by “armored” CAR-T cells can enhance the cells’ migration to the tumor site and counteract immunosuppressive signals, resulting in a more potent and sustained anti-tumor effect.

In conclusion, the “armored” CAR-T cell concept holds great promise for improving the efficacy and durability of CAR-T cell therapy. Further research and clinical trials are needed to verify the efficacy and safety of “armored” CAR-T cell therapy for cancer treatment.

### 6.4 Research and utilization of novel targets

In recent years, research on novel targets has intensified. For example, both CD33 and CD123 are highly expressed in acute myeloid leukemia (AML) cells, providing significant clinical therapeutic effects but also significant toxic side effects.

The transmembrane protein CD317 is a new target antigen against glioblastoma, one of the most aggressive solid tumors. CD317 is not expressed by normal neurons and microglial cells but induces the proliferation of various malignant cells, including liver and breast cancer cells and glioblastoma. A recent study reported the construction of CAR-T cells using lentiviral transduction of CD317. In an *in situ* glioma mouse model, these CAR-T cells showed potent anti-tumor activity, while immunohistochemical staining showed that CD317 was strongly and uniformly expressed in different regions of glioblastoma tissue specimens, in contrast to sections of healthy brain tissue where no or only minimal immunoreactivity was observed, while some organs in the periphery stained positively (73).

Other potential targets include mesothelin (MSLN), a glycophosphatidylinositol (GPI)-linked cell surface protein, that is highly expressed on the surface of mesothelial cells, mesothelioma, and epithelial ovarian cancer cells.

B7 homolog 3 (B7-h3), an immunomodulatory protein, is a recently discovered member of the B7 co-stimulatory and co-inhibitory family of molecules that has been shown to have both immune-activating and inhibitory effects on T-cell-mediated immune responses. While B7-h3 mRNA has not been detected in the nuclei of peripheral blood cells, it has been found in several tumor cell lines with overexpression in a variety of tumors, including brain tumors and pancreatic, ovarian, and gastric cancers. In addition, because B7-H3 is also expressed in tumor stromal cells and tumor stem cells, as well as the tumor neovasculature and other components of the tME, CAR-T cell therapy targeting B7-H3 may be able to destroy tumor cells and modulate the TME. Several studies have reported the construction of mB7-H3, ahB7-H3, and phB7-H3 CAR-T cells, and *in vitro* cytotoxicity comparison experiments have found that ahB7-H3 CAR-T cell therapy resulted in greater specific inhibition of tumor cell growth and cytokine secretion without causing serious toxic side effects, as shown in mouse tests (74). It is a target worth looking forward to.

Glypican-3 (GPC3) is a heparan sulfate proteoglycan that plays an important role in cell growth and differentiation. The function of GPC3 depends on the tissue type. In some tissues, GPC3 acts as a tumor suppressor gene, while in others it functions as a tumor promoter. Studies have shown that GPC3 is a reliable marker for hepatocellular carcinoma, with a sensitivity and specificity exceeding those of the alpha-fetoprotein and hepatocyte paraffin tests. It is thus a promising target for long-term development.

EGFRvIII is a truncated isoform of the epidermal growth factor receptor (EGFR), belonging to a subfamily of receptor tyrosine kinases (HER1–4) mainly expressed in glioblastomas, and has been found to be a key target for organ tumors. EGFR is widely expressed in non-small-cell lung cancer (NSCLC) cell lines and can be used as a target for immunotherapy in these cancers. In a study on EGFR-specific CAR-T cells for the treatment of NSCLC, researchers constructed EGFR-CAR-T cells using genetic engineering and established a subcutaneous tumor model of NSCLC in NSG mice. The results showed that the EGFR-CAR-T cells were effective in inhibiting the growth of NSCLC cells and significantly prolonged the survival of the mice. Thus, the use of EGFR as a target provides a new strategy in the use of CAR-T cell immunotherapy for the treatment of NSCLC (75).

MUC1 is a 200 kDa complex glycoprotein with both transmembrane and secreted isoforms, and is highly expressed in various malignant tumors including esophageal cancer. A recent report describes the design of a CAR-T cell that both targets MUC1 and activates cytokine-cytokine signaling, and verified the effect of this enhanced CAR-T cell therapy on esophageal cancer. The experimental results showed that the enhanced MUC1-CAR-T cells had significant anti-tumor effects on esophageal cancer cells, together with more sustained tumor killing and proliferative capacity compared with conventional MUC1-CAR-T cells. Thus, the use of enhanced MUC1-CAR-T can overcome the limitations of conventional CAR-T cells against solid tumors and provide a new strategy for their applications (76).

In conclusion, different targets have their specific types and characteristics of expression vectors, and the development and utilization of more novel targets will improve the effectiveness of CAR-T cell targeting (77–81) The development and utilization of more novel targets will facilitate the targeting of CAR-T therapy.

### 6.5 CAR-NK cell development

There are various types of immune cells in addition to T cells, including natural killer (NK) and natural killer T (NKT) cells, and even macrophages, which can be used to construct CAR cells. Of these, CAR-NK cells show significant promise. Similar to the concept and general structural design of CAR-T cells, CAR-NK cells consist of extracellular signaling domains, transmembrane regions, and intracellular domains. The safety profile of CAR-NK cells has been found to be superior to that of CAR-T cells in clinical studies as the cytokines produced are usually GM-CSF and IFN-g, which do not belong to the category of cytokines that causes CRS, and therefore the probability of CRS is much lower. In addition, CAR-NK cells have a relatively shorter lifespan during cycling action, thereby reducing toxicity to normal tissue cells expressing the target molecules. Studies have shown that homologous NK cells can be xenografted without causing GvHD, while the low level of PD-1 secreted by NK cells reduces the likelihood of immunosuppression in the TME, suggesting the superior potential of NK cells as a treatment for solid tumors. The sources of NK cells are more diverse, not only through induced pluripotent stem cell production, but also through peripheral blood mononuclear cells derived from umbilical cord blood and other sources. The current drawback to their use is that the CARs that have been designed are tailored for CAR-T cells rather than CAR-NK cells, indicating that the structural design of NK cell-specific CARs requires more in-depth studies to identify suitable stimulatory molecules (82).

One study aims to improve the therapeutic potential of CAR-NK cells through chemical and genetic enhancement. The aim was to develop an innovative approach to enhance the therapeutic effect of CAR-NK cells on solid tumors by utilizing bifunctional lipid nanoparticles (DLNPs) to activate and efficiently deliver mRNA encoding CAR. The study also looked into how DLNPs activate and modulate NK cells, as well as how to optimize the concentration of the cationic lipid 1,2-dioleoyl-3-trimethylammonium propane (DOTAP) in lipid nanoparticles to improve gene delivery efficiency and reduce cytotoxicity. CAR-NK cells targeting Glypcan-3 demonstrated significant therapeutic efficacy in an *in situ* mouse model of hepatocellular carcinoma. These findings highlighted the potential of DLNPs in enhancing CAR-NK cell therapy for solid tumors, representing a significant advancement in NK cell-based cancer immunotherapy and broadening the outlook for NK cell-related disease intervention (83).

In addition, CAR-NK cells show promise for targeting the complex immunosuppressive microenvironment of solid tumors. Till now, researchers have developed a smart biodegradable nanomaterial with peroxidase activity called hollow manganese dioxide nanoparticles (MnOX). This material catalyzes the excess hydrogen peroxide (H_2_O_2_) in the tumor microenvironment to produce oxygen, which helps to alleviate hypoxia in solid tumors. Furthermore, the CD56 antibody was modified onto the surface of the MnOX nanoparticles, enabling it to bind specifically to CAR-NK. The findings revealed that CAR-NK cells bound to MnOX nanoparticles could effectively penetrate tumor tissues and improve the tumor microenvironment, resulting in excellent anti-tumor activity in a solid tumor mouse model. The antibody linkage between the MnOX nanoparticles and CAR-NK minimized the effective dose of MnOX (84).

### 6.6 Combined application of multiple treatments

A clinical trial of patients with malignant tumors showed that patients treated with a combination of CAR-T cell immunotherapy and chemotherapy or radiotherapy had significantly longer overall survival relative to the groups that received chemotherapy or radiotherapy alone. The significance of CAR-T cell immunotherapy in combination with chemotherapy or radiotherapy in improving clinical outcomes is significant. In a mouse model of ROR1-positive NSCLC, after pretreatment with oxaliplatin/cyclophosphamide (Ox/Cy), CAR-T cells showed better penetration of the tumor tissue, resulting in increased chemokine release from tumor-associated macrophages. In addition, the surface expression of PD-L1 in tumor-associated macrophages was significantly increased after combined CAR-T cell therapy and Ox/Cy treatment. Pretreatment with Ox/Cy chemotherapy was found to enhance the migration of CAR-T cells to tumor tissues, while in combination with anti-PD-L1 checkpoint blockade, this treatment regimen enhanced patient survival (85).

Radiotherapy trials using an IFN-γ-dependent approach showed increased levels of adhesion molecules in the TME, thus significantly enhancing the adhesion capacity of T cells (86, 87). Some researchers have combined the advantages of CAR-T cells (strong targeting and ability to cross the blood-brain barrier) with those of SN-38 chemotherapeutic agents (effective vascular penetration, ability to overcome tumor heterogeneity and the TME) at the design level, and prepared SN-38 liposomes using a thin-film-probe supershot method, resulting in the development of a novel CAR-T cell, namely, the SN-38-L-EGFRvIII-CAR-T cell, which not only can exert its own immunocidal effect but can also be used as a drug carrier for the targeted transport of chemotherapeutic drugs, thus combining the antitumor effects of immunotherapy and drug therapy (88). It has good prospects for clinical application.

In addition, because dendritic cells (DCs) are responsible for antigen presentation and the promotion of T-cell infiltration, some researchers have recently tested the therapeutic effects of a DC vaccine combined with MSLN-CAR-T cells, and analyzed the infiltration of CAR-T cells using immunofluorescence. This showed that DC vaccines significantly enhanced the proliferation, infiltration, and persistence of the MSLN-CAR-T cells (89).

In addition to chemotherapeutic approaches, combining radioimmunotherapy and CAR-T cell therapy is a novel strategy for effectively eliminating tumors that do not respond to CAR-T cell therapy. In one study, an NSG mouse model was used, and the tumor was created by implanting tumor cells. The researchers prepared ICAM-1 CAR-T cells and delivered them intravenously to mice. The study also used 177Lu-DOTATATE for radioimmunotherapy. Using SPECT-CT imaging, the researchers observed and assessed the expansion of CAR-T cells as well as their therapeutic effects. The results of the study revealed that combining radioimmunotherapy and CAR-T cell therapy could significantly improve therapeutic efficacy. According to the SPECT-CT imaging results, 177Lu-DOTATATE accurately localized the tumor and calculated the dose absorbed by the tumor during treatment. This study suggests that combining radioimmunotherapy and CAR-T cell therapy can improve tumor efficacy and has the potential to be used to treat tumor types that are resistant to CAR-T cell therapy, but it is limited by the use of human xenogeneic tumor models. Further studies are needed to validate the safety and efficacy of this strategy (90).

### 6.7 Some of the latest prospects

In terms of process engineering for the production of prepared CAR-T cells, a study sought to develop an efficient method for the rapid delivery of CAR mRNA to T cells via lipid-like nanoparticles, thereby increasing the effectiveness of CAR-T cells in cancer treatment. A lipid-like nanoparticle called aLNP (antigenic lipid-like nanoparticle), which contains CD3 and CD28 antibody fragments, was used in the study to activate and expand T cells. First, the CD3 and CD28 antibody fragments were bound to the lipid-like nanoparticles. Then, CAR mRNA was encapsulated in aLNP to ensure efficient transcription into T cells. Finally, the aLNP was co-cultivated with T cells to enable CAR mRNA uptake by T cells. The experimental results demonstrated that aLNP was capable of efficiently delivering CAR mRNA to T cells and causing them to express CAR. Flow cytometry and fluorescence microscopy revealed that CAR-T cells transcribed by aLNP had a high level of activity and anti-tumor ability, and the method was also capable of producing a large number of CAR-T cells in a relatively short period of time (91).

There is a dual-targeting strategy for acute myeloid leukemia (AML). The study combined a novel antibody called AbTCR-CSR with a co-stimulatory receptor (CSR) to create a new cellular platform called “ARTEMIS 2.0.” The AbTCR is the primary signal, while the CSR is the secondary signal. To validate the effect of AbTCR-CSR, the researchers used a variety of experimental methods. Cytotoxicity was measured by co-culturing different leukemia target cells with AbTCR or AbTCR-CSR and analyzing luciferase activity. The study found that AbTCR-CSR effectively killed AML cells. Flow cytometry analysis indicated that AbTCR-CSR showed significant recognition and killing ability in AML cells, but no cytotoxicity in normal peripheral blood mononuclear cells (PBMCs). In addition, AbTCR-CSR recognized and killed other leukemia cell lines and demonstrated anti-tumor activity in a mouse model. This study demonstrates that AbTCR-CSR has significant potential as a dual-targeting strategy for AML treatment. The outstanding feature that AbTCR-CSR can selectively recognize and kill leukemia cells without significant toxicity to normal cells provides an important theoretical basis and experimental foundation for the development of novel therapeutic approaches for AML (92).

In terms of exploring therapeutic efficacy options for solid tumors, some investigators have focused on the development of CAR-T cell therapies targeting tropomyosin-related kinase receptor B (TrkB), a highly expressed protein that is common in many forms of aggressive solid tumors and correlates with disease severity. The researchers screened CAR-T cells for brain-derived neurotrophic factor (BDNF) and neurotrophin 4 (NTF4) ligands, and then used a mouse model to create a tumor model by subcutaneously injecting SMMC7721 cells from a human hepatocellular carcinoma cell line. Non-transduced T cells, or CAR-T cells, were injected into mice through the tail vein five days after the initial injection. Subsequently, the tumor burden was monitored then weekly by biofluorescence imaging. The intervention effect of the T cells was also measured, and the overall condition of the mice was observed by body weight change curves. The experimental results showed that CAR-T cells targeting TRKB were able to significantly reduce tumor burden. Biofluorescence imaging results indicated that the CAR-T cell-treated group had a significantly lower tumor burden than the other groups. Additionally, the researchers observed relatively stable changes in body weight without abnormal fluctuations in the CAR-T cell-treated group of mice. Therefore, CAR-T cells targeting TrkB may be effective in the clinical treatment of solid tumors such as human liver and pancreatic cancers. However, further experimental studies are needed to investigate the specific appropriateness as well as the toxic side effects (93).

## 7 Conclusion

In summary, CAR-T cell therapy represents not only a cutting-edge concept in cell engineering but also a valuable therapeutic program for both research and testing. CAR-T cell therapy is still difficult at present but challenges and opportunities coexist, and as researchers persevere in their research, CAR-T cell therapy is showing rapid development and is gradually demonstrating its application value. The problems associated with CAR-T cell therapy can be divided essentially into the aspects of specificity, durability, safety, and efficacy. Achieving a balance between effectiveness and safety can be difficult. To date, the essential tasks are solving the specific clinical problems and optimizing the design of the program, while poor infiltration, off-target effects, and toxic side effects are the major problems. While cellular immunotherapy has brought significant hope and additional choices to patients with cancer, its high cost is a deterrent for many patients. Improvement of the CAR gene delivery technology represents a way to reduce costs; this includes techniques such as transposon/transposon enzyme-based gene delivery systems, among others. The focus of the next generation of R&D of CAR-T cell therapy will focus on the versatility of the design, as well as reductions in the cost. With the further development of synthetic biology and cellular immunology, CAR-T cell immunotherapy has great potential and bright prospects, and we expect that future developments will enable greater numbers of patients to benefit from CAR-T cell therapy.

## Author contributions

JL: Writing – review & editing, Writing – original draft. XZ: Writing – review & editing, Supervision, Methodology, Funding acquisition.
